# Target antigens for Hs-14 monoclonal antibody and their various expression in normozoospermic and asthenozoospermic men

**DOI:** 10.1186/s12610-015-0025-0

**Published:** 2015-11-06

**Authors:** Jana Capkova, Hasmik Margaryan, Alena Kubatova, Petr Novak, Jana Peknicova

**Affiliations:** Laboratory of Reproductive Biology, Institute of Biotechnology AS CR, the Czech Academy of Sciences, v.v.i., Videnska 1083, 142 20 Prague 4, Czech Republic; Institute of Microbiology AS CR, v.v.i., Videnska 1083, 142 20 Prague 4, Czech Republic

**Keywords:** Acrosome, Human spermatozoa, Monoclonal antibody, Asthenozoospermia, Transitional endoplasmic reticulum ATPase, Acrosome, Spermatozoïdes humaines, Anticorps monoclonal, Asthénozoospermie, Transitoire ATPase de réticulum endoplasmique

## Abstract

**Background:**

Poor semen quality is one of the main causes of infertility. We have generated a set of monoclonal antibodies to human sperm and used them to investigate sperm quality. Some of these antibodies found differences in the expression of proteins between normal sperm and pathological sperm displaying severe defects. One of them was the Hs-14 antibody.

The aim of this paper was to determine the target protein of the Hs-14 monoclonal antibody and to investigate the expression of the Hs-14-reacting protein on the sperm of asthenozoospermic men with sperm motility defect and of healthy normozoospermic men.

**Methods:**

Indirect immunofluorescence, one-dimensional and two-dimensional polyacrylamide gel electrophoresis, immunoblotting and mass spectrometry.

**Results:**

The Hs-14 antibody binds fibronectin, β-tubulin and valosin-containing protein - new name for this protein is transitional endoplasmic reticulum ATPase (TERA). Since the Hs-14 reaction with TERA remained the strongest at the highest antibody dilution, and Hs-14 consistently labelled the same spot or band as the monospecific anti-TERA antibody on immunoblots, we assume that TERA is an Hs-14-specific protein. Binding of fibronectin and β-tubulin might represent nonspecific cross-reactivity or Hs-14 reaction with similar epitopes of these proteins.

A significant difference (*P* < 0.001) in immunofluorescence staining with Hs-14 was found between the normozoospermic and asthenozoospermic men.

**Conclusion:**

The Hs-14 antibody enables discrimination between sterile or subfertile asthenozoospermic and fertile normozoospermic men. Decreased levels of TERA in men can be used as a biomarker of reduced fertility.

**Electronic supplementary material:**

The online version of this article (doi:10.1186/s12610-015-0025-0) contains supplementary material, which is available to authorized users.

## Résumé

**Introduction:**

La pauvre qualité de la semence est l’une des causes d’infertilité. Nous avons généré une série d’anticorps monoclonaux contre le sperme humain et nous l’avons utilisée pour examiner la qualité du sperme. Certains de ces anticorps ont montré des différences d’ expression des protéines entre le sperme normal et le sperme pathologique qui a des défauts sévères. L’un d’eux a été l’anticorps Hs-14. Le but de cet article était de déterminer la protéine cible de l’anticorps monoclonal Hs-14 et d’établir l’expression de la protéine réagissant avec Hs-14 sur le sperme des hommes asthénozoospermiques qui ont des défauts de la mobilité du sperme et sur celui des hommes normozoospermiques.

**Méthodes:**

Immunofluorescence indirecte, electrophorèse sur gel polyacrylamide à une ou deux dimensions, immunoblotting et spectrométrie de masse.

**Résultats:**

L’anticorps Hs-14 s’attache à la fibronectine, à la β-tubuline et à la protéine TERA (ATPase transitoire de réticulum endoplasmique). Etant donné que la réaction du Hs-14 avec TERA a été la plus forte à la dilution la plus grande de l’anticorps, et que Hs-14 marquait systématiquement la même tache ou bande que l’anticorps mono-spécifique anti-TERA sur les immunoblots, nous supposons que TERA est une protéine spécifique pour Hs-14. L’attachement à la fibronectine et à la β-tubuline pourrait représenter une réaction croisée non spécifique ou la réaction du Hs-14 avec des épitopes similaires de ces protéines. Une différence significative (*P* < 0.001) en immunofluorescence avec Hs-14 a été révélée entre hommes normozoospermiques et asthénozoospermiques.

**Conclusions:**

L’anticorps Hs-14 permet de différencier les hommes stériles ou subfertiles asthénozoospermiques des hommes fertiles normozoospermiques. Les niveaux de la TERA chez les hommes pourraient être utilisés comme un marqueur biologique d’une fertilité réduite.

## Background

Antibodies to human sperm proteins and seminal plasma proved to be useful tools for the sperm quality assessment, and consequently for the prognosis of successful fertilization of eggs. In IVF clinics, semen quality is routinely assessed by the concentration, morphology and motility of spermatozoa, as it is given in WHO guidelines [1]. Nevertheless, to better understand the fertility problems, more complex analysis of the expression of individual proteins and their function in the sperm processes, e,g. changes of individual proteins during the acrosomal reaction, capacitation and other processes, is needed [2–7].

Employment of antibodies thus elevated evaluation of sperm ejaculates to the level of investigation of individual proteins relevant for the sperm function. Using the set of monoclonal antibodies that we generated in our laboratory we were able to perform a systematic and continuous analysis of ejaculates for the needs of assisted reproduction. These antibodies can detect poor quality sperm samples even in cases when the parameters of ejaculates meet the requirements of WHO classification for normozoospermics. For example, our antibodies Hs-8, Hs-14 and Hs-36 reliably bound to the acrosomes of spermatozoa in normozoospermic men (60–80 % cells labelled), while their binding was lower in ejaculates with pathological spermiograms (30–40 % cells labelled) [8]. The ability of our anti-acrosomal antibodies to recognize defective spermatozoa was confirmed by assessment of sperm in the mice that were exposed to pollutants [9, 10]. Both findings undoubtedly suggest the importance of the detected proteins in fertilization. The condition when a semen sample complies with the WHO requirements and still is not able to achieve fertilization is not exceptional. In some cases, the expression of certain proteins is altered compared to normal sperm [11] and antibodies represent an appropriate tool to detect the changes in the expression of specific proteins [12]. However, the sperm quality assessment cannot be based on a single protein. Our experience with the Hs-16 monoclonal antibody that detects secretory actin-binding protein (SABP) [13] demonstrated that some normozoospermic samples might display high expression of SABP, whose presence on spermatozoa is associated with sperm pathology [13]. It is obvious that sperm testing must be comprehensive—optimally using a panel of antibodies.

Recently, great possibilities in this respect have been offered by proteomics, where two-dimensional gels enable simultaneous evaluation of hundreds of proteins and provide a complex picture of the investigated sample [14–16].

Comparison between 2D electrophoretic gels of normal and pathological sperm allowed us to select proteins or groups of proteins whose changes in the expression can be a signal of pathological condition [17, 18].

Employment of monoclonal antibodies as a diagnostic tool is dependent on the definition of antibody specificity. The aim of our work was to characterize the Hs-14 monoclonal antibody, i.e., to investigate its target protein and binding in normozoospermic and asthenozoospermic men.

## Methods

### Sperm samples

Human ejaculates were obtained with the participants’ consent from the Centre of assisted reproduction ISCARE I.V.F. (Prague, Czech Republic). All men (of age 25–40 years) gave their written informed consent with donating the sperm ejaculates for the purposes of the research project. The study was also approved by the institutional review board at the Institute of Biotechnology. Thirty sperm samples from men with normal spermiograms and 30 samples from men with asthenozoospermia with reduced motility (<40 %) were assessed. The evaluation of semen density, motility and morphology was carried out in compliance with the World Health Organization standards (2010) [1].

Sperm sample from each men was independently repeated three times. The results were comparable and therefore one of them was chosen for presentation.

### Preparation of Hs-14 monoclonal antibody

The Hs-14 monoclonal antibody (mAb) was generated by standard hybridoma technology introduced to the laboratory by Peknicova et al. [19], after immunization of BALB/c mice with the acid extract of human sperm and fusion with SP2/0-Ag14 myeloma cells (Sigma, Prague, Czech Republic). The acid extract was prepared from the ejaculate of a normozoospermic donor (6 × 10^7^ cells/ml, 60 % motility) as follows: the ejaculate was centrifuged at 200 × *g* and the sperm pellet was resuspended and washed three times in phosphate-buffered saline (PBS, 150 mM NaCl, 17.7 mM NaH_2_(PO_4_).2H_2_O, pH 7.4). The cell pellet was then extracted in 3 % (v/v) acetic acid, 10 % (v/v) glycerol, 30 mM benzamidine for 16 h after cooling at 4 °C with permanent rotation. The extract was dialyzed against 0.2 % acetic acid and lyophilized.

Positive clones were selected by enzyme-linked immunosorbent assay (ELISA) [20] and indirect immunofluorescence test [13, 21]. The Mouse Monoclonal Antibody Isotyping Reagents (ISO-2, Sigma, Prague, Czech Republic) were used to determine the immunoglobulin class of the monoclonal antibody according to the manufacturer’s instructions.

### Antibodies

Besides the Hs-14 monoclonal antibody the following antibodies were used: Prog.13 against progesterone (mouse IgG) prepared in our laboratory [22], TU-06 against the N-terminal domain of β-tubulin (mouse IgM) [23], TU-01 against the N-terminal domain of α-tubulin (mouse IgG_1_) [24] and ab11433 against valosin-containing protein (transitional endoplasmic reticulum ATPase), (mouse IgG, Abcam, UK).

### Purified tubulin

Microtubule protein from porcine brain was prepared according to Shelanski et al. [25]. The entire procedure of tubulin preparation was described in detail by Draber et al. [26]. For preparation of the gel and sodium dodecyl sulphate (SDS) sample of tubulin we used SDS cat.no. L5750 (Sigma, Prague, Czech Republic), which makes possible better separation of α- and β-tubulin.

### Immunocytochemistry

Indirect immunofluorescence was carried out with human spermatozoa. Samples were washed twice with PBS and centrifuged at 200 × *g* for 10 min. Washed cells were diluted in PBS to a final concentration of 2 × 10^7^ cells/ml and 10 μl drops were smeared onto glass slides. Alternatively, spermatozoa were diluted to a final concentration of 1 × 10^6^ /ml and 10 μl drops were loaded on glass slides. Smears or drops were air-dried and then fixed and permeabilized with acetone for 10 min at room temperature (RT, 23 °C). Slides were rinsed in PBS, blocked in PBS-0.05%Tween + 1 % bovine serum albumin + 10 % normal goat serum for 3 h at RT and incubated in a humid chamber with the Hs-14 mAb (undiluted hybridoma supernatant, immunoglobulin concentration <20 μg/ ml) for 60 min at 37 ° C. As a negative control, undiluted supernatant of Sp2/0 myeloma cells was used. After three washes in PBS the slides were incubated with fluorescein isothiocyanate (FITC)-conjugated goat anti-mouse IgM (μ − chain specific) immunoglobulin (Sigma, Prague, Czech Republic) diluted 1:128 in PBS for 1 h at 37 °C. Then the slides were washed in PBS, rinsed in deionized water, quickly air-dried, dropped with mounting medium Vectashield containing DAPI for DNA visualization (Vector Laboratories, Burlingame, CA, USA) and covered with a cover glass. Slides were stored at +4 °C until inspection. In immunofluorescent test 200 cells were evaluated for each sample and each sample was repeated 3 times.

Samples were examined with a Nikon Eclipse E400 fluorescent microscope with Nikon Plan Apo VC oil 60× objective and photographed with CCD camera VDS1300 (Vosskühler, Osnabrück, Germany) with the aid of the NIS elements AR imaging software (Laboratory Imaging, Prague, Czech Republic). Some immunofluorescent samples were also examined with confocal microscope Olympus FV-1000, where digitized images of serial optical 3 μm-thick sections of spermatozoa were collected.

### Electrophoresis, western blotting and immunodetection

Unless otherwise indicated, all chemicals for sample preparation, electrophoresis, blotting and immunodetection were purchased from Sigma (Prague, Czech Republic).

### Sample preparation

In all electrophoretic experiments, samples from normal sperm were used. Ejaculated spermatozoa were washed three times in PBS and used for protein extraction. For one-dimensional polyacrylamide gel electrophoresis (1D PAGE), a dry sperm pellet (1 × 10^8^ cells) was resuspended in 100 μl of non-reducing 2× SDS sample buffer [27] and heated in boiling water bath (3 min). After cooling in + 4 °C and centrifugation (23,100 × *g*, 5 min, 4 °C), the supernatant was divided into aliquots and kept at -80 °C until electrophoresis. To observe the migration of proteins under reducing conditions, samples were supplemented with β − mercaptoethanol (5 % final concentration) and heated for 1 min in boiling water bath prior to electrophoresis.

Sperm cells (1× 10^8^) were resuspended in 100 μl of rehydration buffer (RHB, 7 M urea, 2 M thiourea, 4 % CHAPS, 1 % Triton X-100, 20 mM Tris) or 1 % Triton X-100.

For two-dimensional gel electrophoresis (2D PAGE), sperm cells (1 × 10^8^ cells) were resuspended in 100 μl of RHB and extracted for 1 h at room temperature with occasional shaking. Then the samples were centrifuged (23,100 × *g*, 5 min, RT), and supernatants were aliquoted and stored at −80 °C for subsequent analysis.

### Sodium dodecyl sulphate polyacrylamide gel electrophoresis (SDS PAGE) and Western Blotting (WB)

Polyacrylamide gel electrophoresis in the presence of SDS (SDS PAGE) was performed according to the method of Laemmli [27]. Aliquots of non-reduced or reduced extracts corresponding to 10 μg of protein content were loaded per lane. Proteins were separated in 12 % polyacrylamide gel and visualized by Coomassie Brilliant Blue (CBB-R250, Serva, Heidelberg, Germany) staining or electrophoretically transferred onto PVDF membrane (Immobilon-P Transfer Membrane, Millipore, Bedford, USA), essentially according to the method of Towbin et al. [28]. Molecular masses of the proteins were estimated by comparison with the Precision Plus Protein Standard Dual colour (BioRad, Prague, Czech Republic) running in parallel.

#### Isoelectric focusing

All chemicals, solutions, strips and Ettan IPGphor system for electrophoretic protein separation in the first dimension were purchased from GE Healthcare (Uppsala, Sweden).

The sperm sample, 150 μg (for pH range 3–10) or 200 μg (for pH range 4–7) of protein in total volume of 180 μl of RHB freshly supplemented with 1 % (w/v) dithiotreitol and 2 % (v/v) IPG buffer (pH 3–10, Amersham Biosciences, Uppsala, Sweden), was applied to a 7 cm long strip (Immobiline Drystrip) and passively rehydrated overnight at RT. Proteins were focused for approx. 5 h with 50 mA per strip as follows: 150 V for 50 min, 150–300 V for 1 h (gradient), 300–1000 V for 30 min (gradient), 1000 V for 20 min, 1000–5000 V for 1 h and 20 min (gradient), 5000 V as long as 8000 Vh in total were achieved.

After focusing the strips were incubated in equilibration buffer (EB, 6 M urea, 50 mM Tris–HCl buffer pH 6.8, 30 % (v/v) glycerol, 2 % (w/v) SDS, 0.002 % w/v bromophenol blue), containing 2 % (w/v) dithiotreitol for 15 min and with 1 % (w/v) iodoacetamide in EB for 15 min. Strips were laid onto a 12 % polyacrylamide slab gel for the second-dimension electrophoresis (2D PAGE).

#### Immunodetection

Blots with an identical set of proteins were incubated in PBST solution (0.05 % Tween 20 (v/v) in PBS) supplemented with 3 % gelatin overnight at 4 ° C and afterwards incubated separately for 1 h at RT with undiluted or PBST-diluted hybridoma supernatants (1:1). As a control, the supernatant of Sp2/0 cells (null supernatant) was used. After six 10-min washes in PBST, horseradish peroxidase (HRP)-conjugated goat anti- mouse antibody (GAM/Px, diluted 1:3000, BioRad, Prague, Czech Republic) was applied for 1 h at RT. After that the blots were again intensively washed (1 h, 10 PBST exchanges) and the reaction of antibodies (the corresponding protein bands or spots) was developed with SuperSignal West Pico chemiluminescent substrate (Pierce, Rockford, USA).

Alternatively, the membrane was developed in the dark at room temperature with 0.05 % (w/v) 4-chloro-1-naphtol (Serva, Heidelberg, Germany), 0.001 % (w/v) CoCl_2_ and 0.09 % (v/v) hydrogen peroxide in 0.01 M Tris–HCl (pH 7.4). The reaction was stopped after 10 min by washing the membrane in distilled water.

Relative molecular masses of proteins detected by monoclonal antibodies were estimated by comparison with the mobility of molecular mass protein standards running in parallel after staining of blots with Coomassie Brilliant Blue (0.25 % CBB R250, 7 % CH3COOH, 50 % ethanol).

### Mass spectrometric analysis

#### CBB staining

Gels intended to mass spectrometry were incubated for 1 h at room temperature in a solution containing CBB-R250, and in the solution of 35 % ethanol, 10 % CH3COOH until the background disappeared and the separated proteins in 2D PAGE were clearly and sharply visible.

#### Sample preparation and proteolytic digestion

All chemicals for sample preparation and mass spectrometric analysis were purchased from Sigma-Aldrich (St Louis, MO, USA).

CBB-R250-stained protein spots of interest were excised from the gel and decolorized several times in sonic bath at 60 °C with 10 mM dithiothreitol, 0.1 M 4-ethylmorpholine acetate (pH 8.1) in 50 % acetonitrile (ACN) until complete destaining. Then the gel was washed with water, shrunk by dehydration with ACN and reswollen by incubation in 60 mM iodoacetamide, 0.1 M 4-ethylmorpholine acetate (pH 8.1) for half an hour in the dark at RT. After cysteine alkylation, the gel was washed with water, shrunk by dehydration with ACN and reswollen in water. The rehydration and dehydration of the gel was repeated three times. Next, the gel was reswollen in 0.05 M 4-ethylmorpholine acetate (pH 8.1) in 50 % ACN and partly dried using a SpeedVac concentrator (Savant, Holbrook, NY, USA). Finally, the gel was reconstituted with cleavage buffer containing 0.01 % 2-mercaptoethanol, 0.05 M 4-ethylmorpholine acetate, 10 % ACN, and sequencing grade trypsin (Promega, 50 ng/μl). Digestion was carried out overnight at 37 °C, and the resulting peptides were extracted with 30 % ACN/0.1 % trifluoroacetic acid and subjected to mass spectrometric analysis.

#### MALDI/FT-ICR MS (Matrix Assisted Laser Desorption/Ionization Fourier Transformed Ion Cyclotron Resonance Mass Spectrometry)

Masses of individual peptides obtained after tryptic digestion of Hs-14-detected protein(s) were determined by the peptide mass fingerprint. Mass spectra of peptides were measured using a MALDI/FT-ICR mass spectrometer (9.4 T APEX-Qe, Dual ion source II, Bruker Daltonics, Billarica, U.S.A.), a peptide map(s) was (were) established, and mass spectra were searched against the human Swissprot database using Mascot 2.3 software (Matrix Science). The spectrum was calibrated internally using the monoisotopic [M + H]^+^ ions of trypsin autoproteolytic products. A saturated solution of α-cyano-4-hydroxy-cinnamic acid in 50 % ACN/0.2 % TFA was used as a MALDI matrix. One μl of matrix solution was mixed with 1 μl of the sample on the target and the droplet was allowed to dry at ambient temperature. The mass accuracy was set to less than 3 ppm for the data interpretation.

#### Statistical analysis

Experimental data were analysed using GraphPad Prism 5.04. The differences between the normozoospermic and asthenozoospermic group in the number of Hs-14-positive cells were analysed by two-tailed Mann Whitney test. The p value equal to or lower than 0.05 was considered to be significant, *p ≤ 0.05, **p ≤ 0.01, ***p ≤ 0.001.

## Results

### Hs-14 monoclonal antibody

By fusion of immune spleen cells with Sp2/0 myeloma cells we obtained 80 independently derived clones. One of the positive clones, D5A12, generated the hybridoma producing monoclonal antibody designated Hs-14. Analysis of immunoglobulin class showed that the Hs-14 antibody belongs to IgM molecules.

### Immunofluorescent localization of the antigen recognized by the Hs-14 mAb

**Fig. 1:**
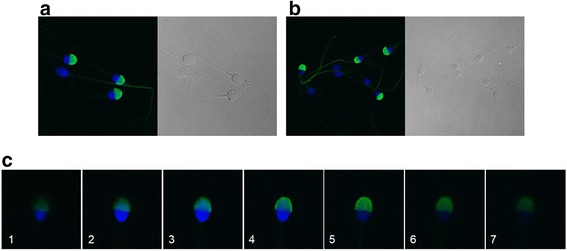
Immunostaining of representative samples of human ejaculated spermatozoa with Hs-14 monoclonal antibody and corresponding phase-contrast microphotographs. The antibody strongly labelled sperm acrosome (**a**, **b**). In some samples, apart from the acrosome, the flagellum of spermatozoa was also stained (**b**). Pictures of a series of 3-μm distributed sections of sperm cell in confocal microscope. The 3rd to the 5th sections show the highest staining (**c**). Blue colour – DAPI staining of the nucleus, green colour – FITC staining (Fig. 1a and 1b magnification 600×, Fig. 1c magnification 1000×)

Indirect immunofluorescence of samples of spermatozoa with Hs-14 mAb demonstrated intensive staining of acrosomes. The Hs-14 mAb strongly labelled the acrosome in 60–90 % ejaculated spermatozoa after permeabilization of the cell membrane of samples from normozoospermic men (Fig. 1a, 1b). The immunofluorescence showed that the antibody also labelled the flagellum (Fig. 1b). This heterogeneous labelling was not visible in all tested samples. The staining of the flagellum was obvious only in a low number of cells or not at all. The intra-acrosomal localization of the Hs-14-corresponding protein was confirmed by examination under confocal laser scanning microscope. Digitized images of serial optical 3 μm-thick sections of spermatozoa showed the most intensive staining from the 3rd to the 5th section (Fig. 1c). The samples from asthenozoospermic men showed the same staining, but the numbers of sperm were lower (not shown).

### Western blot analysis of human sperm protein(s) detected by the Hs-14 mAb

**Fig. 2:**
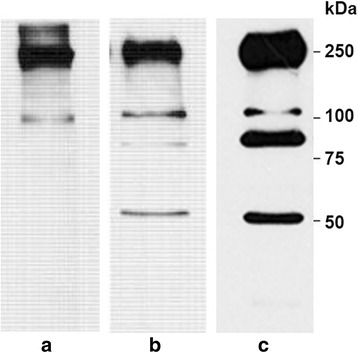
Immunoblots of human sperm extracts with Hs-14 mAb. On WB with SDS extract the antibody recognized a protein band of about 240 kDa and a weak protein band of about 110 kDa under non-reducing conditions (**a**). Under reducing conditions, two new bands of about 95 and 55 kDa appeared (**b**). On WB with RHB extract the antibody recognized the same protein bands (**c**). In some reduced extracts the band of about 55 kDa was lacking (not shown). Molecular mass markers (Mr × 10^3^) are shown in the margins

The pattern of immunoreactive human sperm proteins observed in Western blots of 12 % gels is shown in Fig. 2. Under non-reducing conditions the Hs-14 mAb showed a strong signal at high molecular weight at approximately 240 kDa (p240) (Fig. 2a). In most Western blots a weaker reaction of Hs-14 with a protein band of approximately 110 kDa (p110) was also observed. When β-mercaptoethanol (2-ME) was used to reduce disulphide bonds, protein bands with mobility corresponding approximately to 240, 110, 90–97 and 50–55 kDa appeared (Fig. 2b). The same results were observed with RHB sperm extracts (Fig. 2c). Control Western blots incubated with supernatant of Sp2/0 myeloma cells (null supernatant) and with an antibody of irrelevant specificity (Prog.13, anti-progesterone) displayed no immunoreactive proteins (not shown).

### Identification of the Hs-14-corresponding proteins by MALDI mass spectrometry

**Fig. 3:**
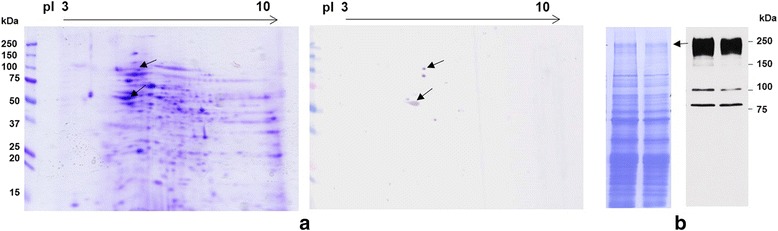
2D electrophoresis in RHB of human sperm extract and immunoblot with Hs-14 mAb (**a**). Isoelectric focusing was carried out in pH 3–10, and the separation in the second dimension was run in 12 % polyacrylamide gel. On the corresponding blot the Hs-14-reacting proteins are indicated by arrows and were subjected to mass spectrometry. In some cases the Hs-14 mAb reacted on WB also with proteins of about 240 and 110 kDa, but these proteins had no counterparts in 2D gels. Therefore, the 240 kDa band for MS was selected from SDS PAGE according to the corresponding immunoblot (**b**)

To identify proteins reacting with the Hs-14 mAb on Western blot, corresponding protein spots were found on 2D gel (Fig. 3a), excised and subjected to MALDI/FT-ICR MS. Mass spectra of peptides were measured and searched against the Swissprot database using Mascot software. Two proteins were identified by peptide mass fingerprint as TERA and β-tubulin. The protein corresponding to TERA was independently identified two times (from two different gels) and the sequence coverage was 40.8 and 55.8 %, respectively. The theoretical molecular weight (90 kDa) and isoelectric point of 5.0 fit well the experimental 2D PAGE data. The sequence coverage for β-tubulin was 62.7 %. The theoretical molecular weight (50 kDa) and isoelectric point of 4.6 fit well the experimental 2D PAGE data as well. The Hs-14 reaction with another two proteins—of about 240 and 110 kDa—was visible after 2D electrophoresis on some Western blots only, nevertheless, their counterparts were not found on 2D gels. Therefore, these two proteins were analysed by MALDI/FT-ICR MS from the 1D gel (Fig. 3b). The protein of about 240 kDa was determined as a fibronectin precursor with sequence coverage 24.7 %, theoretical molecular weight 266 kDa and isoelectric point 5.4. The protein of about 110 kDa could not be identified. Identification of TERA by MALDI mass spectrometry is presented in Additional file 1: Figure S1.

### Comparison of anti-TERA and Hs-14 reaction with human sperm extract

**Fig. 4:**
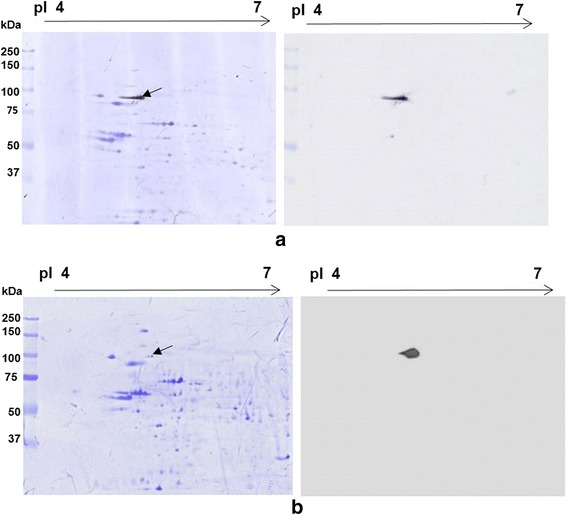
2D electrophoresis in RHB of human sperm extract and immunoblot with antibody Hs-14 (**a**) and immunoblot with anti-TERA mAb (**b**). The protein spot reacting with antibody is marked on CBB-stained PVDF membrane with an arrow

Recognition of TERA by the Hs-14 mAb was verified using commercial anti-VCP (anti- TERA) monoclonal antibody. On the immunoblot with human sperm extract separated by 2D PAGE, the Hs-14 antibody (Fig. 4a) labelled the same spot as anti-TERA antibody (Fig. 4b).

**Fig. 5:**
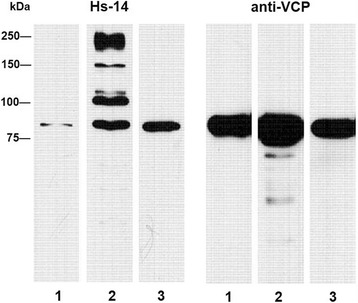
Reactions of Hs-14 antibody and anti-TERA antibody with different human sperm extracts RHB (columns 1, 2) and Triton X-100 (column 3) on immunoblot. These different sperm extracts were two times loaded on the same electrophoretic gel. After western blotting the membrane was divided into two halves and at each half were given two different antibodies (Hs-14, TERA)

Nevertheless, the Hs-14 reaction and the anti-TERA reaction were not quantitatively identical. The Hs-14 mAb reacted on blots with TERA more weakly than the monospecific anti-VCP antibody. However, the anti-TERA antibody reacted with differing intensity with various human sperm extracts and the Hs-14 mAb copied the intensity of reactions with anti-TERA (Fig. 5).

### Reaction of Hs-14 and anti-tubulin mAbs with purified tubulin

**Fig. 6:**
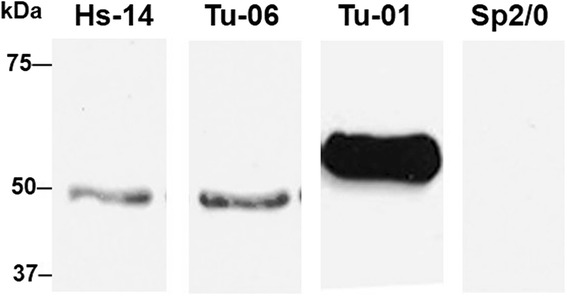
Reaction of monoclonal antibodies Hs-14 and anti-tubulin antibodies (TU-06 and TU-01), and supernatant Sp2/0 (negative control) on Western blot with purified tubulin. Hs-14 and TU-06 antibodies bound the N-terminal domain of β-tubulin

Recognition of tubulin with the Hs-14 mAb was confirmed by reaction with purified tubulin. The purified tubulin was separated electrophoretically on 7 % polyacrylamide gel, and on Western blot with the transferred tubulin the Hs-14 and TU-06 antibodies reacted identically. More precisely, they bound to the β-tubulin subunit while the TU-01 antibody reacted with α-tubulin and had no counterpart in the Hs-14 reaction (Fig. 6).

### Immunodetection of the human sperm proteins with diluted Hs-14 mAb

**Fig. 7:**
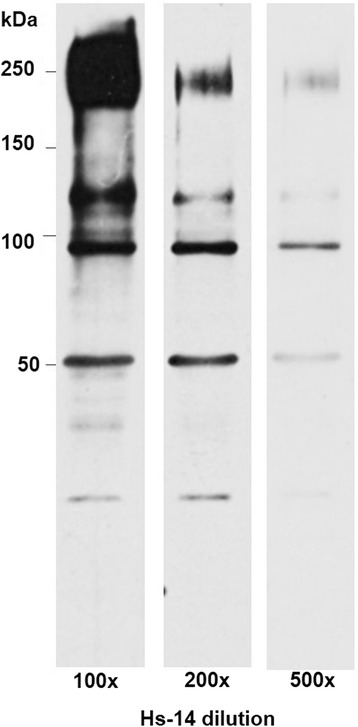
Immunodetection of human sperm proteins with diluted Hs-14 mAb. At the highest Hs-14 antibody dilution (1:500), the reaction with 95 kDa protein (TERA) was the strongest

At the highest dilution of the Hs-14 antibody (Hs-14 supernatant, 1:500), the strongest antibody reaction on Western blot with separated RHB human sperm extract was again with the 95 kDa protein that was determined by MALDI/FT-ICR MS as TERA. The reaction with the approximately 110 kDa protein band completely disappeared and reactions with the bands of approximately 240 and 50 kDa were very weak (Fig. 7).

### Expression of the Hs-14-reacting protein(s) in the sperm of normozoospermic and asthenozoospermic men

**Fig. 8:**
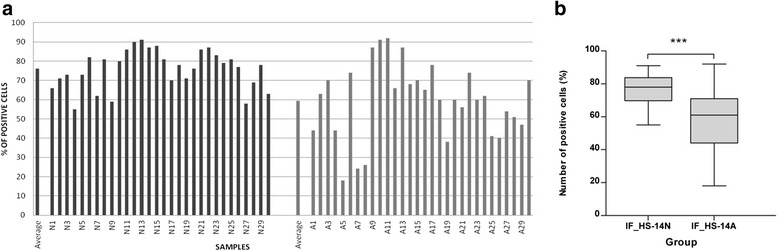
Hs-14 mAb-labeled spermatozoa in 30 sperm samples originated from men with normal spermiograms (N) and 30 samples from men with asthenozoospermia (A) Columns represent the average percentage of spermatozoa with Hs-14 labelling of acrosome, each sample was evaluated three times. (**a**). The difference in the number of Hs-14-positive cells between men with normozoospermia and asthenozoospermia was evaluated by two-tailed Mann Whitney test and was significant (*p* ≤ 0.001) (**b**). Middle lines indicate the arithmetic means, bars denote the standard deviations (SD), and whiskers indicate minimum and maximum of the values

Permeabilized spermatozoa of 30 individual samples from the groups of both normozoospermic and asthenozoospermic men displayed different expression of the Hs-14 reacting protein(s) in indirect immunofluorescence (Fig. 8). Asthenozoospermic men had a significantly lower count of sperm labelled with the Hs-14 antibody compared to normozoospermic men. The statistical difference was evaluated by two-tailed Mann Whitney test and was significant at 0.1 % level (*P* value < 0.001).

## Discussion and conclusion

The Hs-14 monoclonal antibody was proved successful in the assessment of human semen quality [8, 9]. A significant positive correlation was found between the Hs-14-detected protein(s) on the sperm and the semen quality.

To characterize the antibody we applied indirect immunofluorescence, biochemical methods and mass spectroscopy. Further, using the Hs-14 antibody we investigated the relevant proteins on the sperm from men with normozoospermia and asthenozoospermia.

Based on our results, we can conclude that the Hs-14 monoclonal antibody is not monospecific but binds several proteins or similar epitopes of these proteins. After one-dimensional electrophoresis, the antibody recognized four protein bands of about 240, 110, 95 and 50 kDa on the immunoblot. After sperm sample separation in two-dimensional electrophoresis, two protein spots reacting with the Hs-14 mAb were found on the blot. Molecular masses of these spots were about 95 and 55 kDa, and mass spectrometric analysis identified these proteins as transitional endoplasmic reticulum ATPase (TERA) and tubulin, respectively. These results were obtained repeatedly with different samples. The two remaining proteins—of 240 and 110 kDa—were not identified on the gel after 2D electrophoresis. One of these proteins, of 240 kDa, was extracted from the gel after 1D electrophoresis and sequenced, and was identified as a fibronectin precursor. Nevertheless, protein sequencing from the 1D gel has limitations.

The participation of these three proteins in fertility has already been investigated and their role was found to be highly significant.

Fibronectin (Fn) is an adhesive molecule that binds to β1, 3 and 4 integrins on the sperm surface [29] and immunofluorescence revealed a broad distribution pattern of Fn on the sperm heads [30]. In human sperm, Fn has various functions: it activates the proteasome and induces the acrosomal reaction [31], participates in the sperm-egg interaction [32], and increased Fn concentration is associated with decreased sperm motility and thus with fertility problems in men [33, 34].

Tubulin is the main structural element of the cell cytoskeleton. In mature spermatozoon tubulin is localized in two major compartments: flagellum and head [26, 35, 36]. The flagellum is composed of a typical arrangement of microtubules and is the organ of sperm motility [35], which is closely related to sperm fertility. Our previous study revealed differences in the amount of β-tubulin among men with normozoospermia and pathological spermiograms [37].

Transitional endoplasmic reticulum ATPase (TERA) is a glycoprotein that is involved in membrane fusion and presentation of ubiquitinated proteins to the proteasome [38]. TERA is found in the sperm of different species and is a substrate of cAMP-activated sperm tyrosine kinase [39]. In human sperm TERA is one of the proteins that are phosphorylated during capacitation [40]. Recently, TERA was identified as one of the targets of post-translational modification, sumoylation in the sperm [41]. Sumoylation gives rise to small ubiquitin-like modifiers (SUMO) that are implicated in the regulation of numerous cellular events and also in the maturation and differentiation of sperm [41].

Thus, each of these proteins can affect the sperm ability to fertilize eggs. We tried to find whether Hs-14 specifically recognizes all the three proteins that were identified by mass spectrometry and is polyspecific or whether the Hs-14 reactivity is partially caused by non-specific cross-reactivity or the reaction with similar epitopes of these proteins. Some characteristics of the relevant proteins indirectly exclude specific reaction with the Hs-14 antibody. The concentration of fibronectin on the sperm surface is negatively correlated with the sperm motility [33, 34], while the number of spermatozoa labelled with Hs-14 is positively correlated with the sperm quality. Immunofluorescent examination of spermatozoa by confocal microscopy also demonstrated intracellular localization of the target protein, while fibronectin is a spermadhesive protein.

The mass spectrometry determination of tubulin recognition by Hs-14 was verified by direct reaction of Hs-14 with purified tubulin on Western blot and β − tubulin was confirmed as one of the Hs-14-reacting proteins. However, in immunofluorescence the antibody labelled the flagellum, where tubulin is located, less often than in the acrosome or not at all. This knowledge also supports non-specific Hs-14 reaction with tubulin.

The Hs-14 specificity was also investigated after its dilution. At 500× dilution, the antibody reacted considerably more strongly with TERA than with β-tubulin and fibronectin. This reaction indicated TERA as the most probable Hs-14-specific protein, while Hs-14 reaction with β-tubulin and fibronectin suggested nonspecific cross-reactivity.

In addition, the reaction of monoclonal anti-TERA and Hs-14 antibodies with various independently prepared sperm extracts indicates TERA as the Hs-14 specific protein. Both antibodies consistently labelled the same band of about 95 kDa on Western blots. Only in the case of the strongest sperm extract (Fig. 7) Hs-14 labelled additional bands.

We also wanted to identify the Hs-14 target protein by immunoprecipitation of human sperm lysate with the Hs-14 antibody. For the experiment we used a direct immunoprecipitation kit (Pierce Protein Biology product) according to the manufacturer’s instructions, in which the relevant antibody (Hs-14) was covalently bound to aldehyde-activated beaded agarose resin and sperm protein lysate was added. Unfortunately, we did not obtain an unequivocal result. The following electrophoresis did not determine one protein only and it also supported Hs-14 nonspecific cross-reactivity with several proteins. Similar results were obtained by a classical immunoprecipitation test with protein A.

The other reason for the difficulty in unequivocally identifying the Hs-14 target protein can be the complex IgM nature of the Hs-14 antibody. IgM antibodies are frequently associated with unspecific cross-reactivity, and therefore are generally less reliable.

As mentioned above, the basic aim of this study was to identify the Hs-14-recognized protein(s), because the antibody proved to be a good marker for sperm quality evaluation [8]. In our previous investigations we followed the differences among normozoospermic and pathological sperm mainly with severe damage. Here we utilized the antibody and concentrated on the differences between normozoospermic and asthenozoospermic men found by the Hs-14 antibody. Spermatozoa of asthenozoospermic men are seemingly of good quality, with normal morphology, and the sperm concentration in asthenozoospermic ejaculates is also normal (>15 × 10^6^ cells per ml) [1]. The only apparent defect is their decreased motility. Still, many asthenozoospermic patients are sterile or subfertile and this deficiency can be circumvented by in vitro fertilization using intracytoplasmic sperm injection.

Monitoring of asthenozoospermic and normozoospermic samples showed decreased Hs-14 protein expression in 15 out of 30 asthenozoospermic men (<60 % labelled cells), while only three out of 30 followed normozoospermic individuals displayed less than 60 % labelled cells. Statistical analysis showed a significant difference (*P* < 0.001) in the number of Hs-14- positive spermatozoa between both groups.

Our data showed decreased expression of relevant sperm protein TERA (formerly VCP) in asthenozoospermic men. Recently, TERA was identified as a protein playing a role in ubiquitination [41]. Decreased detection of this protein in asthenozoospermic men can most probably be explained by spontaneous acrosome reaction, which may occur in ejaculated sperm or impaired synthesis of the protein in the sperm with pathological spermiograms.

## Conclusion

Our data suggest transitional endoplasmic reticulum ATPase (TERA - formerly VCP) as target protein of Hs-14 monoclonal antibody and revealed decreased expression of TERA in asthenozoospermic men in comparison with normozoospermic ones. We can conclude that appropriate monoclonal antibodies against sperm can be used not only as biomarkers of sperm quality, but also for detection of sperm defects at the molecular level.

## Additional file

Additional file 1: Figure S1. MALDI/FT-ICR MS analysis of CBB-R250staining spot – peptide map of TERA. (PPTX 224 kb)
