# Supplementary figures and images for: Target antigens for Hs-14 monoclonal antibody and their various expression in normozoospermic and asthenozoospermic men
Source: Basic Clin Androl. 2015 Nov 6;25:11. doi: 10.1186/s12610-015-0025-0 (PMC4636759; doi:10.1186/s12610-015-0025-0)

## Slide 1
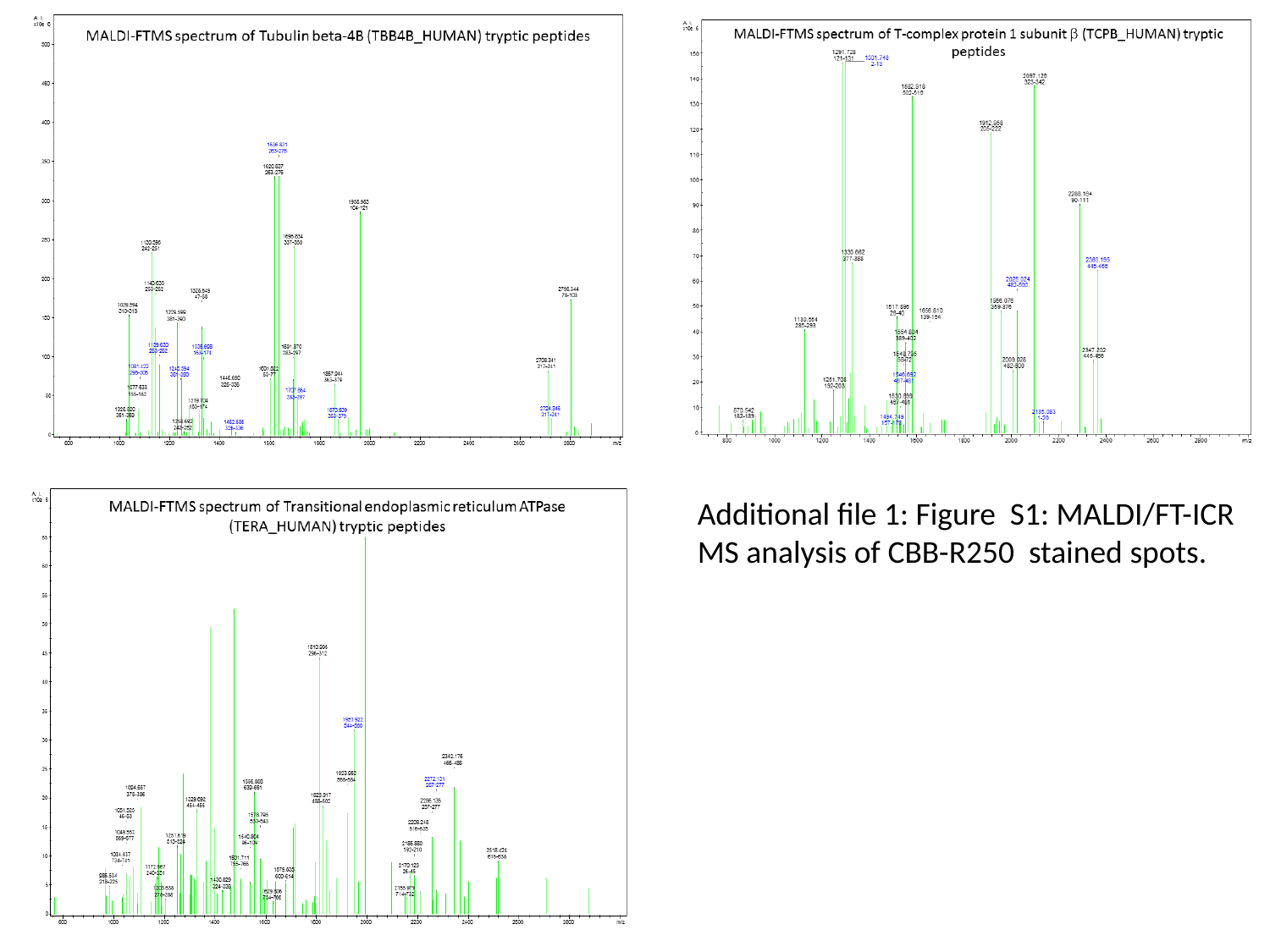

Additional file 1: Figure S1: MALDI/FT-ICR MS analysis of CBB-R250 stained spots.

Supplement: Additional file 1: Figure S1. — MALDI/FT-ICR MS analysis of CBB-R250staining spot – peptide map of TERA. (PPTX 224 kb) [file 12610_2015_25_MOESM1_ESM.pptx]
